# Pro-angiogenic photo-crosslinked silk fibroin hydrogel: a potential candidate for repairing alveolar bone defects

**DOI:** 10.1590/1678-7757-2023-0158

**Published:** 2023-08-28

**Authors:** Siyuan Wu, Xuezhong Zhou, Yilong Ai

**Affiliations:** 1 Foshan University Foshan Stomatology Hospital School of Medicine Foshan China Foshan University, Foshan Stomatology Hospital & School of Medicine, Foshan, China.

**Keywords:** Silk fibroin, Hydrogel, Vascularization, Alveolar bone defect

## Abstract

**Objective:**

This study aimed to develop a pro-angiogenic hydrogel with in situ gelation ability for alveolar bone defects repair.

**Methodology:**

Silk fibroin was chemically modified by Glycidyl Methacrylate (GMA), which was evaluated by proton nuclear magnetic resonance (1H-NMR). Then, the photo-crosslinking ability of the modified silk fibroin was assessed. Scratch and transwell-based migration assays were conducted to investigate the effect of the photo-crosslinked silk fibroin hydrogel on the migration of human umbilical vein endothelial cells (HUVECs). *In vitro* angiogenesis was conducted to examine whether the photo-crosslinked silk fibroin hydrogel would affect the tube formation ability of HUVECs. Finally, subcutaneous implantation experiments were conducted to further examine the pro-angiogenic ability of the photo-crosslinked silk fibroin hydrogel, in which the CD31 and α-smooth muscle actin (α-SMA) were stained to assess neovascularization. The tumor necrosis factor-α (TNF-α) and interleukin-1β (IL-1β) were also stained to evaluate inflammatory responses after implantation.

**Results:**

GMA successfully modified the silk fibroin, which we verified by our 1H-NMR and *in vitro* photo-crosslinking experiment. Scratch and transwell-based migration assays proved that the photo-crosslinked silk fibroin hydrogel promoted HUVEC migration. The hydrogel also enhanced the tube formation of HUVECs in similar rates to Matrigel^®^. After subcutaneous implantation in rats for one week, the hydrogel enhanced neovascularization without triggering inflammatory responses.

**Conclusion:**

This study found that photo-crosslinked silk fibroin hydrogel showed pro-angiogenic and inflammation inhibitory abilities. Its photo-crosslinking ability makes it suitable for matching irregular alveolar bone defects. Thus, the photo-crosslinkable silk fibroin-derived hydrogel is a potential candidate for constructing scaffolds for alveolar bone regeneration.

## Introduction

Alveolar bone defects refer to bone loss in the tooth-supporting alveolar process of the jaw, which can be caused by various factors such as periodontal disease, trauma, or tooth extraction.^1,2^ Alveolar bone defects can lead to significant functional and aesthetic problems and can compromise the success of dental implantation and other restorative procedures.^3^ Vascularization plays a crucial role in alveolar bone regeneration as it provides the necessary oxygen and nutrients for cell growth and tissue repair.^4-6^ Without adequate vascularization, bone regeneration can be slowed down or even halted altogether.^7,8^ This can lead to complications such as delayed healing, non-union, or even the development of bone necrosis. Thus, strategies enhancing vascularization could be promising treatments for alveolar bone defects.

Several strategies have been developed to enhance vascularization in alveolar bone regeneration, including the use of growth factors and cell-based therapies.^9^ Growth factors, such as the vascular endothelial growth (VEGF) and basic fibroblast growth factors (bFGF), have been shown to promote bone regeneration angiogenesis and neovascularization.^10-12^ Cell therapies such as mesenchymal stem cells (MSCs) can secrete pro-angiogenic factors and differentiate into endothelial cells, contributing to the formation of new blood vessels.^13-15^ Reports also suggest that the exosomes of MSCs also promote the growth of new vessels during bone regeneration.^16,17^ Although growth factor and cell-based therapies showed promising therapeutic efficacy, controlling quality is complicated and their sources are usually unstable.^18-20^ Thus, new strategies are still in need to address this problem.

Compared to growth factors and cell-based therapies, biomaterials offer easier quality control and greater accessibility and affordability.^21^ Biomaterials can also provide a three-dimensional structure to support cell growth and tissue formation.^22^ Moreover, biomaterials can be designed to match the geometry and mechanical properties of the damaged tissue, providing a precise fit and minimizing any negative effects on the surrounding tissue.^22^ Furthermore, their composition can be tailored to match the biological and mechanical properties of the native tissue, which can help to promote tissue integration and minimize the risk of rejection or adverse reactions.^23^ Hence, pro-angiogenic biomaterials are suitable for alveolar bone repair.

Silk fibroin (SF) is a natural protein fiber obtained from silkworm, *Bombyx mori*, cocoons. Due to its good biocompatibility and biodegradability, the FDA (U.S. Food and Drug Administration) has recognized silk fibroin as a safe and effective biomaterial for medical devices and drug delivery systems.^24^ In fact, several medical devices based on silk fibroin have already received FDA approval and are commercially available.^25^ Besides, SF has been reported to be anti-inflammatory.^26-28^ As prolonged or severe inflammatory response will hinder bone regeneration,^29-31^ SF might benefit bone regeneration. Thus, developing pro-angiogenic biomaterial products based on silk fibroin might be a promising strategy for repairing alveolar bone defects.

This study obtained a photo-crosslinked SF hydrogel by chemically modifying SF. Compared with conventional methods for preparing silk fibroin-derived hydrogel, such as ultrasound, ethanol, and horse radish peroxidase/H_2_O_2_ treatments, photo-crosslinking makes the gelation process efficient (averaging seconds^32^) and enables *in situ* formation, thus matching the irregular shape of alveolar bone defects. Moreover, as vascularization is a critical process for bone regeneration, we conducted both *in vitro* and *in vivo* experiments to evaluate the pro-angiogenic ability of the photo-crosslinked silk fibroin-derived hydrogel to determine whether it configures a potential candidate for repairing alveolar bone.

## Methodology

### Synthesis of MeSF and LAP

Methacrylated silk fibroin (MeSF) was synthesized based on recent studies.^32,33^ A 40-g unit of raw silk fiber (Bombyx mori) was boiled at 100 °C for 30 h in 2 L of 0.05 M Na_2_CO_3_ for degumming. The degummed silk was washed and dried at room temperature (RT). Then, 100 mL of 9.3 M lithium bromide (LiBr) solution was used to dissolve 15 g of dried silk at 60 °C for 1 h, 15 mL of a glycidyl methacrylate (GMA) solution was added dropwise, and the mixture was stirred at 60 °C for 3 h. Right after the reaction was complete, the mixture was cooled to RT and dialyzed against distilled water using 8-14 kDa cutoff dialysis tubes for four days. Lastly, the GMA-modified SF was frozen at −80 °C for one night. After lyophilization for five days, the dry MeSF sponges were stored at −20°C for use.

Lithium phenyl(2,4,6-trimethylbenzoyl) phosphate (LAP) was synthesized following a recent study.^34^ Briefly, 3.2 g 2,4,6-trimethylbenzoyl chloride (Sigma-Aldrich) was reacted with 3.0 g Dimethyl phenylphosphonite at RT under argon gas. After stirring the reaction mixture for 18 h, 6.1 g LiBr in 100 mL of 2-butanone was added into it and the reaction system was heated to 50 °C. After 10 minutes, the reaction system was cooled to RT for 4 h and the reaction mixture was filtered to harvest the solid precipitate. The filtrate was then washed by 2-butanone several times and dried in a vacuum.

### Characterization of MeSF (NMR)

To confirm the synthesis of MeSF, proton nuclear magnetic resonance (^1^H-NMR) was used at 600MHz with a DPX FT-NMR Spectrameter (Bruker, Germany). For sample preparation, 600 μL of deuterium oxide (D_2_O, Sigma-Aldrich) were used to dissolve the 5-mg MeSF/SF sponges. MeSF and SF spectra were recorded. By comparing the spectra of MeSF and SF, the methacrylation of SF was further confirmed.

### Preparation of precursor solutions

Dried MeSF sponges were dissolved in PBS (simultaneously mixing LAP) to obtain solutions (15% wt/v). LAP was used at a unit of 1% of the weight of MeSF sponges. After the mixture was totally dissolved, the precursor solutions were stored at 4 °C for further use.

### Preparation of MeSF-conditioned medium

In total, 1 mL of MeSF precursor solution was crosslinked by 405-nm light in 10-cm cell culture dish and 10 mL of L-DMEM (Gibco^®^) was added. Then, this system was incubated at 37 °C for 24 h. Finally, fetal bovine serum (FBS) (Gibco^®^) and penicillin/streptomycin (PS) (Gibco^®^) were added at 10% and 1% volume ratio, respectively, to obtain the MeSF-conditioned medium.

### CCK-8 assay

In total, 100-μL human umbilical vein endothelial cells (HUVECs, ATCC^®^ Cat. No. PCS-100-010™) (density: 1. 10^4^ cells/mL) or 100-μL L929 (NCTC clone 929) suspensions (density: 1. 10^4^ cells/mL) were added into each well of a 96-well plate. The medium was changed after 24 h of seeding. The wells were divided into two groups; one group was treated with complete L-DMEM and the other, with MeSF-conditioned complete L-DMEM. The CCK-8 assay (DOJINDO) was conducted on day 1, 3, and 5 and guided by the manufacturer's instruction book.

### Scratch assay

A 2-mL human umbilical vein endothelial cells (HUVECs, ATCC^®^ Cat. No. PCS-100-010™) suspension (density: 4 . 10^5^ cells/mL) was added into each well of a 6-well plate and the cells were grown to confluence with complete L-DMEM in 2 days. The monolayer was then scratched with a sterile pipette tip or a scalpel to create a gap with a uniform width. The cells were washed to remove any debris and fresh media was added to the dish. The dish was then placed under a microscope and the gap was imaged at regular intervals to monitor its closure rate. The migration and proliferation of cells into the gap could be quantified by measuring the change in gap width over time. The control group was treated with complete L-DMEM and the MeSF group, with MeSF-conditioned complete L-DMEM, as illustrated in the section “Preparation of MeSF-conditioned medium.”

### Transwell migration assay

The MeSF precursor solution was added to the bottom of 24-well cell culture plate and crosslinked to form a coating layer. The control group plate was given no treatment. Then, complete L-DMEM was added into it. The transwell inserts (pore size 8 μm) were put into a 24-well plate and 200 μL of the HUVECs suspension (4 . 10^5^ cells/mL) were added into the transwell inserts. After 24 h incubation, the non-migrated cells on the upper surface were gently removed with a cotton swab and washed with PBS. Next, the migrated cells were fixed by 4% paraformaldehyde (PFA) for 15 minutes at room temperature. Finally, the migrated cells were stained by DAPI (Beyotime), imaged by fluorescence microscope, and quantified with Image J.

### QRT-PCR

**Figure 1:**
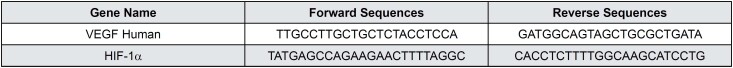
Angiogenic genes

In total, three wells of the 6-well plate were coated with 1 mL MeSF hydrogel and 2 mL of the human umbilical vein endothelial cells (HUVECs, ATCC^®^ Cat. No. PCS-100-010™) suspension (density: 4 . 10^5^ cells/mL) was added into each well of a 6-well plate. After 24 h, the medium was cleaned and the cells were collected using TRIzol (500 μl for each well) (Thermo fisher). The extracted RNA was reverse-transcribed with a Revert Aid first-strand cDNA synthesis kit (Takara, Shiga, Japan) according to the manufacturer's instructions. Then, RT-PCR was conducted using the ABI Step One Plus real-time PCR system (Applied Biosystems, USA) and SYBR Green RT-PCR kit (Takara, Japan) to detect the expression of target genes (Figure 1). Each procedure was repeated thrice for each sample and the ΔΔcT method was used to detect the level of relative gene expression.

### *In vitro* angiogenesis assay

The wells of a 96-well plate were coated with 50 μl of nothing (control group), MeSF hydrogel, and Matrigel 354230 (Corning^®^, a basement membrane matrix derived from the Engelbreth-Holm-Swarm mouse sarcoma used as a positive control). The Matrigel 354230 solidified when incubated at 37°C for 30 minutes. Then, 100 μl of the HUVECs suspension (4 × 10^5^ cells/mL) were added to each well of the 96-well plate. The plate was incubated at 37°C in a humidified incubator with 5% CO_2_ for 72 hours. The cells were imaged using an inverted microscope at set time intervals. The formation of tube-like structures by the cells was analyzed using Image J 1.52q1.52v.

### Subcutaneous implantation

Healthy male rats were selected for experiments (∼250 g). Isoflurane inhalation was used as anesthesia. The skin on the back of the rats was shaved and sterilized. A small incision was made by a scalpel and a pocket was created using scissors to accommodate the implant. After that, the MeSF hydrogel (10 mm diameter and 2 mm height) was put into the pocket and the incision was closed using sterile sutures. After a week, the rat was euthanized to harvest the implant and the surrounding tissue. The tissue was imaged using smartphones and fixed by 4% PFA for two days at room temperature for further study.

### H&E staining

First, distilled water was used to wash all samples to remove PFA. All samples were embedded in paraffin and sliced into thin sections (5 μm thickness). Finally, the sections were deparaffinized, hydrated, and stained by hematoxylin and eosin.

### Immunofluorescence staining

For immunofluorescence (IF) staining, sectioned slides (n=3 per group) were randomly chosen. At first, all slides were deparaffined with xylene and hydrated with graded ethanol and distilled water. Then, all slides were blocked with 5% (wt/v) bovine serum albumin (BSA, Sangon Biotech, China) solutions at RT for 1 h. The blocked slides were then incubated with primary rabbit/mouse specific antibodies (α-SMA (Abcam, ab32575), CD31 (Abcam, ab222783), IL-1β (Abcam, ab254360), and TNF-α (Abcam, ab66579) at 4°C overnight. Then, the slides were incubated using Alexa Fluor^®^ 488 or 546 (1:500, Invitrogen) conjugated secondary antibodies at RT for 2h to visualize these proteins.

### Statistical analysis

All experiments were performed with at least three replicates. Results are all shown as means ± SDs. *P* values lower than 0.05 (*p<0.5, **p<0.05, ***p<0.01, ****p<0.0001) were considered to be statistically significant. Data were analyzed by GraphPad Prism 8 (GraphPad Software, Inc, USA) using one-way ANOVA.

## Results

### Synthesis and characterization of MeSF

**Figure 2:**
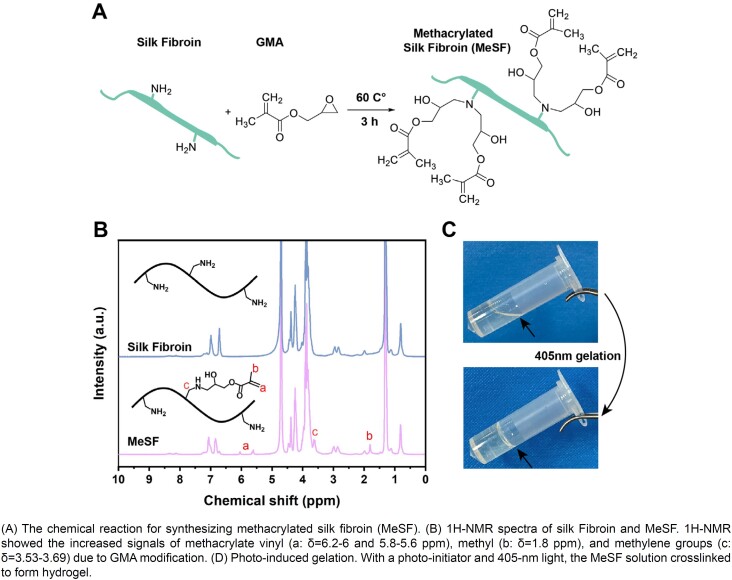
Synthesis and characterization of MeSF

Figure 2A shows the synthesis of the glycidyl methacrylate (GMA)-modified silk fibroin (MeSF). We conducted ^1^H-NMR to evaluate SF methacrylation (Figure 2B). We found methyl (δ=1.8 ppm) and methacrylate vinyl groups (δ=6.2-6 and 5.8-5.6 ppm) from GMA in the MeSF samples, meaning that we had successfully modified SF with GMA. Then, we prepared a MeSF solution, using Lithium phenyl(2,4,6-trimethylbenzoyl) phosphate (LAP) as a photo-initiator. We employed UV light at 405nm to treat the aforementioned solution for 10s and crosslink it, transforming it into a stable gel (Figure 2C). These results suggested that we successfully synthesized MeSF and that it could crosslink under UV exposure.

### MeSF-conditioned medium promoted the migration of HUVECs

**Figure 3:**
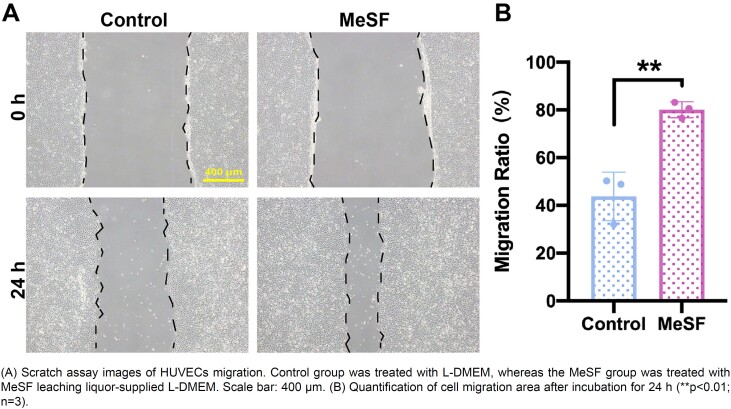
The MeSF-conditioned medium promoted the migration of HUVECs with scratch assay

**Figure 4:**
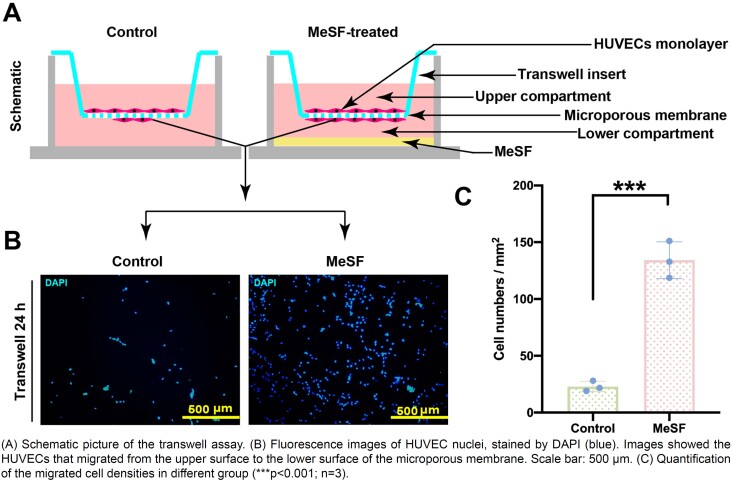
The conditioned medium of MeSF promoted the migration of HUVECs with transwell assay

To test whether MeSF had the ability to promote vascularization, we conducted two *in vitro* experiments. As the migration of endothelial cells is crucial for the formation of new vessels, we firstly used the MeSF-conditioned medium and scratch assay to assess the effect of MeSF on the migration pattern of HUVECs. By comparing it to the normal medium, results show that the MeSF-conditioned medium significantly promoted the migration of HUVECs (Figure 3). To further validate our findings, we designed a transwell-based experiment to investigate the effect of MeSF hydrogel on the migration of endothelial cells. As the schematic picture illustrates, we used the MeSF hydrogel at the lower compartment of the transwell and the HUVECs seeded on the upper surface of the microporous membrane to evaluate whether MeSF hydrogel could affect the migration of HUVECs by calculating the number of transferred HUVECs from the upper to the lower surface (Figure 4A). After 24 h-treatment, we counted the number of migrated HUVECs. Results agreed with the scratch assay, i.e., the MeSF-conditioned medium significantly promoted the migration of HUVECs (Figure 4B, C). The CCK-8 assay also proved that the MeSF-conditioned medium promoted the proliferation of HUVECs (Figure S1). Hence, we proved that MeSF can promote the migration and proliferation of endothelial cells.

### MeSF promoted the tube formation of HUVECs

**Figure 5:**
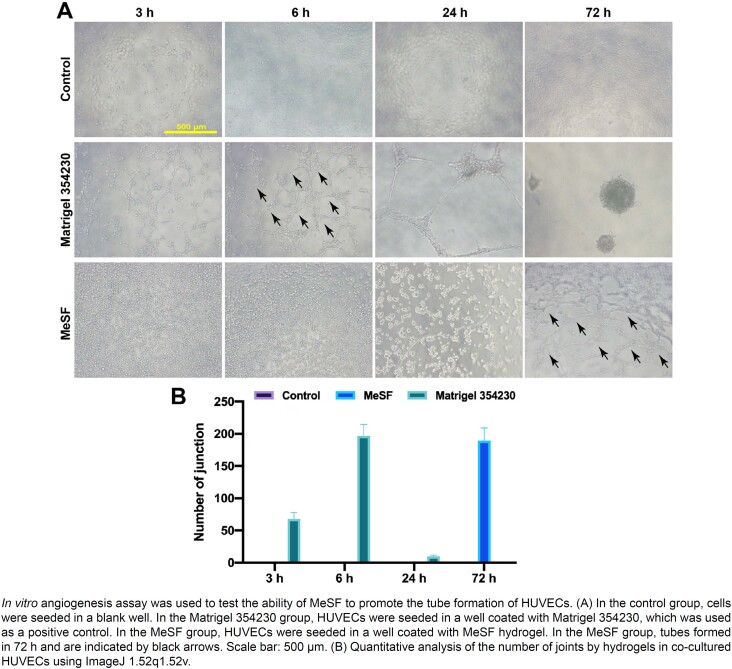
MeSF promoted the tube formation of HUVECs *in vitro*

To investigate whether endothelial cells could form a tube structure on MeSF, we conducted an *in vitro* angiogenesis assay with a commercial Matrigel® (Matrigel 354230) as a positive control. We seeded the HUVECs different surfaces, blank a plate (control), the Matrigel 3542307, and MeSF hydrogel (Figure 5). Results indicated that HUVECs successfully formed tubes on Matrigel within six hours as Matrigel contains abundant factors that could promote vascularization (Figure 5). However, qRT-PCR results proved that after 24 h of incubation, the angiogenic genes were upregulated in MeSF groups (Figure S2). Thus, when we seeded cells on MeSF hydrogel we obtained tube structures (although this required more time). However, we observed no tube structure in control group (Figure 5).

### MeSF promoted *in vivo* neovascularization

**Figure 6:**
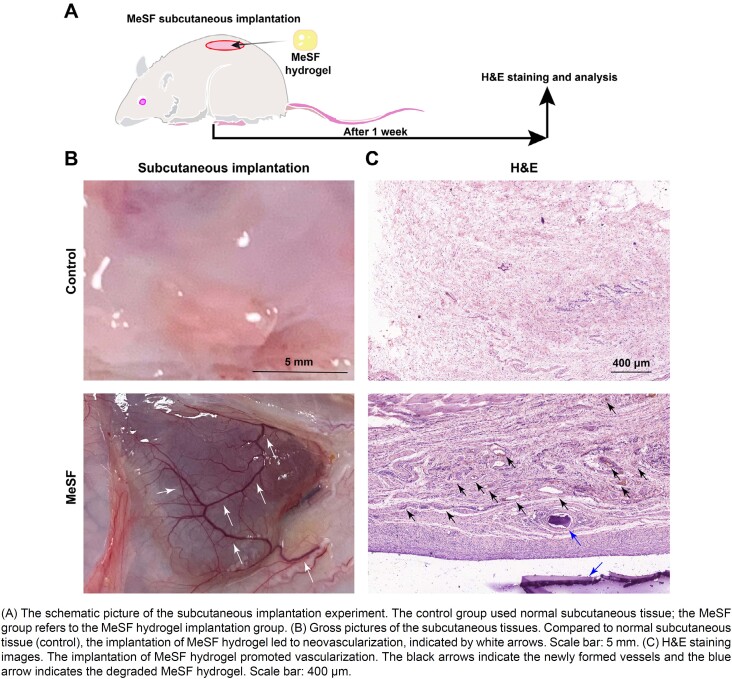
MeSF promoted vascularization *in vivo*

The CCK-8 assay firstly proved that the MeSF showed no cytotoxicity (Figure S3). Thus, we used *in vivo* a subcutaneous implantation model to further validate the pro-angiogenic ability of the MeSF hydrogel (Figure 6A). After one week of implantation, we harvested the hydrogel and the surrounding tissue. Compared to normal subcutaneous tissue, we observed abundant newly formed vessels surrounding the MeSF hydrogel (Figure 6B). We found no tissue necrosis at the implantation site. Next, we embedded and sectioned the samples for H&E staining to further observe the degree of neovascularization. The H&E staining results showed hydrogel pieces and plenty of vessels surrounding the MeSF hydrogel and little vessels in normal subcutaneous tissue (Figure 6C).

### MeSF promoted *in vivo* neovascularization and inhibited inflammation

**Figure 7:**
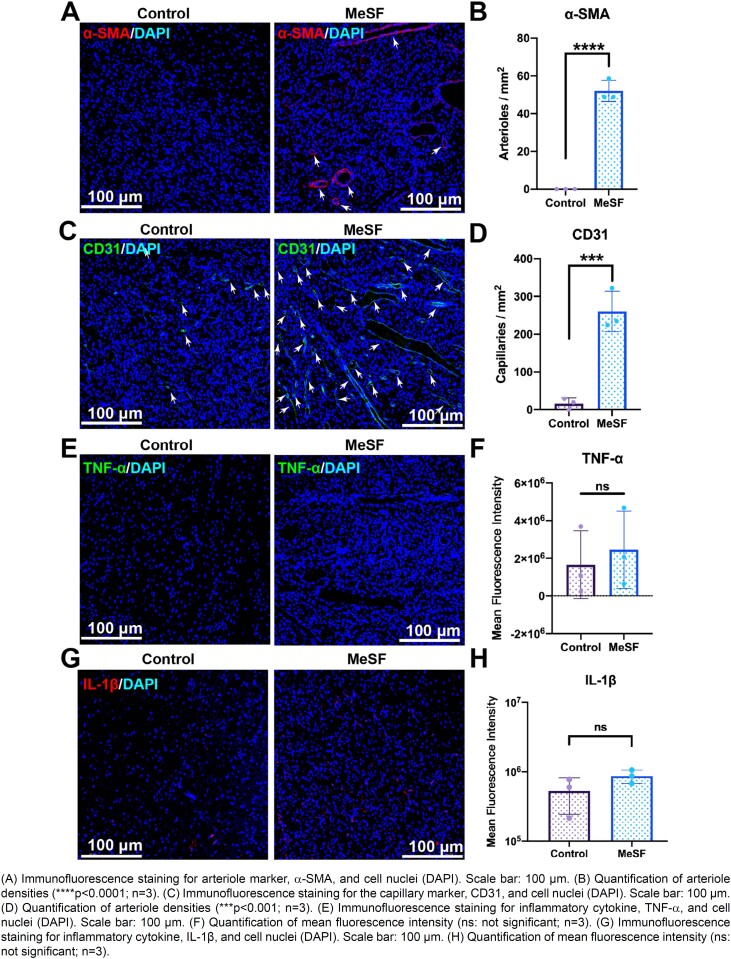
MeSF promoted vascularization and inhibited inflammation *in vivo*

Moreover, we used immunofluorescence (IF) staining to test the expression level of inflammatory cytokine, tumor necrosis factor-α (TNF-α), interleukin-1β (IL-1β), and vascularization markers (α-smooth muscle actin (α-SMA) and CD31). Firstly, the IF staining results of α-SMA and CD31 proved that the implantation of MeSF hydrogel induced both arterioles and capillaries formation (Figure 7A-D). Besides, the IF staining results of inflammatory cytokine TNF-α and IL-1β proved that MeSF triggered no severe inflammatory response at the implantation site (Figure 7E-H). Together, the aforementioned results indicate that the implantation of MeSF hydrogel could promote neovascularization without triggering inflammatory response. Thus, MeSF hydrogel is a promising potential biomaterial for tissue regeneration.

## Discussion

Vascularization is a crucial process for alveolar bone regeneration. Tissue engineering commonly use stem cells, growth factors, and biomaterials to promote vessel formation.^6-8^ However, compared with stem cells, cell products, and growth factors, biomaterials show easier quality control and scalable production.^21^ Besides, the biomaterial is a crucial component for engineered bone scaffolds, which provide mechanical supports and porous structures for cell migration.^22^ Thus, pro-angiogenic biomaterials are more promising for constructing scaffolds to promote alveolar bone regeneration. We obtained a silk fibroin-derived photo-crosslinked hydrogel with pro-angiogenic ability. *In vitro*, the MeSF hydrogel promoted the migration of HUVECs. Besides, compared with HUVECs seeded on Matrigel, HUVECs seeded on MeSF hydrogel successfully formed tube structures. Moreover, MeSF components are chemically clearer, rendering it much more suitable for tissue engineering and scalable production. *In vivo*, the MeSF hydrogel enhanced neovascularization within one week after subcutaneous implantation. Note that the implantation of biomaterials might trigger inflammatory reactions. Although these reactions may also promote vascularization, they usually lead to scar formation instead of functional tissue regeneration.^31,35,36^ According to our results, the implantation of MeSF induced no severe inflammatory response, as indicated by the expression level of TNF-α and IL-1β at the wound site. Together, the MeSF hydrogel could promote vascularization without inducing a severe inflammatory reaction at the implantation site, which is beneficial to tissue regeneration, such as bone regeneration.

Our findings also show that biomaterials could be used to modulate the key biological process at the wound site. Liang et al. proved that, compared with alginate-derived hydrogel, silk fibroin-derived hydrogel recruited specific cell populations at the wound and modulated key biological processes, leading to better healing outcomes.^33^ Besides, Elisseeff et al. showed that synthetic and natural materials induced different inflammatory responses after implantation, which also led to different regeneration outcomes of muscle tissue.^37-40^ In summary, recent research brought a conception that biomaterials could modulate the biological processes at the implantation site. Our research also proved that MeSF favored vascularization, which suggests that MeSF could be used as a modulator for neovascularization to affect tissue regeneration.

In clinical cases, alveolar bone defects are usually irregular, making it difficult for scaffolds to match their shapes.^1^ The photo-crosslinking ability of MeSF assures *in situ* gelation. In other words, the MeSF could be stored as liquid and added to the defects using syringes. After that, the frequently used ultraviolet light in dental department could be used to crosslink the MeSF solution. Thus, due to *in situ* gelation, the MeSF hydrogel could perfectly match the shape of irregular bone defects. In summary, the photo-crosslinking ability of MeSF makes it a promising biomaterial candidate for irregular alveolar bone defects regeneration.

Limitations of this study mainly lie in the mechanism of our findings. Although we found a useful feature of a silk fibroin-derived and photo-crosslinked hydrogel, mechanism investigation of the pro-angiogenic ability of MeSF is insufficient. Many studies have shown that biomaterial components, 3D structure, and kinds could affect angiogenesis.^41-43^ However, in our scratch, transwell, and CCK-8 assays, the leaching liquor of the MeSF hydrogel or MeSF hydrogel-conditioned medium promotes the migration of HUVECs. Thus, we speculate that the degraded fragments of the MeSF affect the behavior of endothelial cells, leading to neovascularization. Thus, we will use RNA-sequencing techniques and other high-throughput techniques to investigate its underlying mechanism. Besides, the biocompatibility of MeSF hydrogel should be systematically evaluated for use in clinical cases. Finally, our team is also interested in using MeSF to repair critical alveolar bone defects, hoping to validate our conclusion that MeSF could promote bone regeneration by enhancing neovascularization.

## Conclusion

In this study, we synthesized a photo-crosslinkable precursor that could be used to obtain silk fibroin-derived hydrogel. Our findings proved that the MeSF hydrogel promoted both *in vitro* and in *vivo* vascularization. More importantly, inflammatory reactions were inactive during the vascularization period. Besides, the photo-crosslinking ability of MeSF makes *in situ* gelation possible, which means it easier to match the shape of irregular alveolar bone defects. Thus, these advantages make MeSF a promising biomaterial candidate for alveolar bone defects regeneration.

## Data Availability

The datasets generated and analyzed during the current study are available in the SciELO Data repository, https://doi.org/10.48331/scielodata.YKRIKZ.
